# Immunotherapy for the treatment of multiple myeloma

**DOI:** 10.3389/fimmu.2022.1027385

**Published:** 2022-10-28

**Authors:** Leora S. Boussi, Zachary M. Avigan, Jacalyn Rosenblatt

**Affiliations:** ^1^ Department of Medicine, Beth Israel Deaconess Medical Center, Boston, MA, United States; ^2^ Division of Hematology, Department of Medicine, Beth Israel Deaconess Medical Center, Boston, MA, United States

**Keywords:** multiple myeloma, immunotherapy, CAR T therapy, adoptive cell therapy, cancer vaccines

## Abstract

Despite advances in treatment for multiple myeloma, the majority of patients ultimately develop relapsed disease marked by immune evasion and resistance to standard therapy. Immunotherapy has emerged as a powerful tool for tumor-directed cytotoxicity with the unique potential to induce immune memory to reduce the risk of relapse. Understanding the specific mechanisms of immune dysregulation and dysfunction in advanced myeloma is critical to the development of further therapies that produce a durable response. Adoptive cellular therapy, most strikingly CAR T cell therapy, has demonstrated dramatic responses in the setting of refractory disease. Understanding the factors that contribute to immune evasion and the mechanisms of response and resistance to therapy will be critical to developing the next generation of adoptive cellular therapies, informing novel combination therapy, and determining the optimal time to incorporate immune therapy in the treatment of myeloma.

## Introduction

Multiple myeloma (MM) is a plasma cell malignancy characterized by clonal proliferation of terminally differentiated B cells in the context of an immunosuppressive milieu that permits immune escape (1, 2). There have been remarkable treatment advances for both front-line and relapsed and refractory (R/R) disease that have dramatically improved survival in recent years, including the use of anti-CD38 monoclonal antibody therapy, proteasome inhibitors, and immunomodulatory imide drugs. However, current therapies are not curative, with 5-year survival less than 60%, and most patients ultimately relapse with resistant disease (3–5). As such, a critical area of recent discovery lies in novel immunotherapeutic approaches to target the malignant plasma cell clone and overcome the immunosuppressive tumor microenvironment, which has the potential to treat previously refractory disease and create a more durable response to therapy. Here, we will review mechanisms of immune dysregulation in MM as well as current advances in immune and cellular immunotherapy, including adoptive cell transfer, T cell engagers, checkpoint inhibition, and vaccine therapy.

## Immune dysregulation in myeloma

Immune dysfunction and evasion in myeloma is mediated by multiple cytokine and cellular signaling pathways which decrease immune effector cell function and create a suppressive bone marrow microenvironment. Immune dysregulation in the bone marrow is driven in part by soluble components such as transforming growth factor (TGF)-β, interleukin (IL)-10, IL-6, and prostaglandin E2, which are produced by the malignant plasma cell and by other suppressive cell populations in the tumor microenvironment, including regulatory T cells, myeloid derived suppressor cells (MDSCs), and bone marrow stromal cells (6). Additionally, upregulation of negative checkpoint pathways, including the PD-1/PD-L1 pathway, contributes to impaired T cell mediated killing of the malignant clone. PD-L1 is highly expressed on myeloma cells, and PD-1 has been shown to be upregulated on circulating T cells isolated from patients with advanced MM (7). These and other pathways drive T cell dysfunction, including impaired cytotoxic T lymphocyte activation, reduced CD4 T cells, increased T regulatory cells, and hallmarks of T cell exhaustion, which have been described even in monoclonal gammopathy of undetermined significance (MGUS) (6, 8, 9). Critical aspects underlying this process include T cell senescence and ineffective antigen presentation by tumor and dendritic cells (DCs) (9–12). Indeed, DCs isolated from MM patients are decreased in number and profoundly dysfunctional (6, 13). Factors contributing to DC dysfunction include IL-6–mediated inhibition, decreased expression of human leukocyte antigen (HLA), high levels of PD-L1 expression, and decreased expression of co-stimulatory molecules (6, 10, 11). Innate immunity is further impaired by suppression of NK cell function by MM and tumor evasion of NK cell surveillance (14). Corresponding changes in the surrounding microenvironment include increased presence of immunosuppressive cells such as regulatory T cells, regulatory B cells, MDSCs, tumor-associated macrophages (TAMs), and mesenchymal stem cells (MSCs) (6, 15, 16). In particular, increased presence of MDSCs has been associated with worse outcomes in MM (16–18).

As understanding of these complex interactions disrupting immune equilibrium has expanded, immunotherapy has emerged as a strategy to overcome the immunosuppressive tumor microenvironment and promote host anti-tumor immunity in myeloma (19). These efforts have focused on the activation and expansion of effector cells which can target and attack malignant cells as well as creation of a durable memory response to confer long term remission and protection against tumor relapse (20).

## Allogeneic stem cell transplant

The potential role of immunotherapy for MM was initially established with the graft-versus-myeloma effect of allogeneic hematopoietic cell transplantation (allo-SCT). Allo-SCT has been shown to induce long-term remission and potential cure in a subset of MM patients, which is thought to be due to anti-myeloma activity of graft alloreactive lymphocytes (2, 21–23). The efficacy of this approach was further underscored by success of donor lymphocyte infusion (DLI) for patients who relapsed after allo-SCT (24). However, significant toxicities including infection, graft-versus-host disease (GVHD), and treatment-related mortality have limited its use, particularly with the introduction of newer induction and maintenance regimens in MM leading to improved response rates (25). To reduce treatment-related morbidity and mortality and increase the pool of allo-SCT eligible patients, non-myeloablative or reduced-intensity conditioning (RIC) allo-SCT has similarly been used (25–27); however, this strategy has been associated with increased risk of relapse, highlighting the importance of initial high-dose cytotoxic conditioning for durable response and remission (2, 25). High-dose chemotherapy with autologous transplant followed by RIC allo-SCT has been evaluated to potentially balance these two approaches, but studies have demonstrated some late relapses as well as unclear efficacy or reduced survival when compared with tandem autologous transplantation (2, 23, 25). While allo-SCT may still have a potentially curative role in a curated subset of patient (28), risks related to infection and GVHD remain significant. Nonetheless, the potency of the graft-versus-myeloma effect speaks to the unique potential for the immune system to overcome resistance, and fuels the field of immunotherapy which strives to more specifically target the malignant plasma cell clone.

## Adoptive cellular therapy

### CAR T therapy

Chimeric antigen receptor (CAR) T cell therapy has demonstrated dramatic response rates in patients with advanced myeloma, resulting in the FDA approval of two CAR T cell products for patients with R/R disease (idecabtagene vicleucel and ciltacabtagene autoloeucel). CAR T cells are generated by transducing autologous T cells with the CAR construct, consisting of an antigen binding site, generally a single variable chain targeting a tumor antigen expressed on the malignant clone, a costimulatory domain, and the ζ-chain of the T cell receptor (TCR) complex. Upon ligation, the receptor signals through the ζ-chain of the TCR complex as well as a costimulatory molecule, most commonly CD28 or 4-1BB, to induce CAR T expansion and anti-tumor immunity through targeted lysis of malignant cells (29, 30). The inflammatory potential, persistence, and toxicity of the CAR T construct are impacted by choice of costimulatory signaling (29, 30). Specifically, CD28 is associated with a terminal effector cell phenotype with more rapid expansion, robust cytokine production, and early-onset CRS, while 4-1BB pushes cells toward a memory phenotype with slowed expansion, but with increased persistence and reduced exhaustion (31–34). CAR T toxicity can include on-target/off-tumor effects on non-malignant cells expressing the antigen of interest. More commonly, toxicity is driven by dysregulated immune hyperactivation and cytokine production, including TNFα, IL-6, and IL-1α, which lead to cytokine release syndrome (CRS), a syndrome of systemic inflammation characteristically including fever and capillary leak, as well as neurotoxicity (34).

In MM, CAR T cells were initially studied targeting a variety of antigens, including CD19, CD138, light chains, NKG2D, and the Lewis Y antigen, with mixed results (35–40). Specifically, initial efforts to target a population of myeloma-propagating cells using an anti-CD19 CAR T product concurrently with autologous transplant significantly increased progression-free survival (PFS) in a small subset of responders (35, 36). However, the most robust and promising outcomes in patients with R/R disease have been shown with CAR constructs targeting B-cell maturation antigen (BCMA), with key trials highlighted in **Table 1**. BCMA is a member of the tumor necrosis factor superfamily expressed preferentially on mature B cells that is crucial for survival of plasma cells and has limited expression on hematopoietic stem cells or other tissues, making it an ideal therapeutic target (47, 48). An initial trial of 16 patients with R/R myeloma treated with an anti-BCMA CAR T clone using a CD28 costimulatory domain showed an overall response (OR) rate of 81%; however, this construct induced high rates of toxicity, with 94% of patients developing CRS and 38% grade 3 or above (41). To reduce toxicity, idecabtagene vicleucel (ide-cel) was developed with a 4-1BB costimulatory domain (42, 43). In a phase 2 study of 128 patients with R/R myeloma who had received at least 3 prior therapies, patients treated with ide-cel had an OR of 73%, complete response (CR) of 33%, and PFS of 8.8 months (43). While patients had high rates of CRS overall (84%), only 5% developed grade 3 or above CRS, and 18% of patients developed neurotoxicity including just 3% with grade 3 or higher reactions, suggesting a more tolerable toxicity profile. Notably, CAR T clones persisted for at least 12 months in 36% of patients, suggesting the potential for prolonged disease control (43).

**Table 1:** Selected published and ongoing clinical trials of CAR T cells in myeloma.

Phase	Pts	CAR construct	Clinical Response	Toxicity (overall, severe)	Ref.
1	16	BCMA, CD28	OR: 81%; VGPR/CR: 63%; median EFS: 31 wks	CRS: 94%, 38% Neuro: 0%	(41)
1	33	Ide-cel (bb2121); BCMA, 4-1BB	OR: 85%; CR: 45%; median PFS 11.8 mo.	CRS: 76%, 6% Neuro: 42%, 3%	(42)
2	128	Ide-cel (bb2121); BCMA, 4-1BB	OR 73%; CR 33%; median PFS 8.8 mo.	CRS: 84%, 5% Neuro: 18%, 3%	(43)
1b/2	97	Cilta-cel; BCMA (two domains), 4-1BB	OR: 97%; CR 67%; 1-year PFS 77% (median not reached at 12.4 mo.)	CRS: 95%, 3% Neuro: 21%, 9%	(44)
1	9	OriCAR-017; GPRCD	OR: 100%; CR/sCR 38%	CRS: 100%, 0% Neuro: 0%	(45)
1	72	bb21217; BCMA, 4-1BB, PI3K inhibitor	OR: 69%; CR/sCR 28%; median duration of response 17 mo.	CRS: 75%, 4% Neuro: 15%, 4%	(46)

OR, overall response; VGPR, very good partial response; CR, complete response; EFS, event-free survival; PFS, progression-free survival; sCR, stringent complete response; CRS, cytokine release syndrome.

To increase avidity of the CAR T clone, ciltacabtagene autoleucel (cilta-cel) was designed with two distinct BCMA-targeting antibodies as well as a 4-1BB costimulatory domain (44). In a multicenter phase 1/2 trial of 97 patients with R/R myeloma, cilta-cel showed an OR of 97% and CR of 67%. Significantly, 1-year overall survival (OS) was 89% and PFS 77%, with median duration of response not reached at median follow up of 12.4 months; further studies are ongoing for long term outcomes. The toxicity profile was similar to ide-cel, with high incidence of CRS (95%) but low rates of severe disease (4%), and similar rates of neurotoxicity (21% overall, 9% severe) (44). Finally, other targets have been identified including G-protein coupled receptor, class C group 5 member D (GPCR5D), a hair follicle protein that is upregulated in myeloma cells with a similar distribution to BCMA (49). Preliminary results from an ongoing phase 1 study of an anti-GPRC5D CAR OriCAR-017 (NCT05016778) were recently presented in 9 patients with R/R myeloma, including patients previously treated with anti-BCMA CAR T therapies. Initial data showed a 100% OR rate, with 100% incidence of CRS but 0 grade 3 events (45).

Despite their transformational role in treatment of R/R disease and the potential for durable remission, emergence of CAR T resistance remains a significant challenge. Multiple mechanisms of CAR T resistance have been described, including emergence of antigen-negative clones, immune clearance or poor survival of the CAR T cells, and CAR T cell exhaustion (50–53). In MM specifically, biallelic loss of BCMA expression has been shown in a subset of patients with recurrence after CAR T therapy (54, 55). One method to combat BCMA loss is the use of γ-secretase inhibitors to increase membrane BCMA expression. γ-secretase cleaves membrane-bound BCMA to allow shedding from plasma cells, and the resulting soluble BCMA can further limit CAR T cell recognition of myeloma cells (56, 57). A phase 1 study of the γ-secretase inhibitor JSMD194 in conjunction with anti-BCMA CAR T cells is currently underway (NCT03502577). Another strategy to reduce effects of antigenic loss on efficacy is development of CAR T cells simultaneously targeting multiple tumor antigens, such as BCMA with CD38 (58, 59), CS1 (60), or GPRC5D (49, 61, 62), as well as a novel approach of an anti-BCMA CAR with a CD38 chimeric costimulatory receptor to further drive lymphocyte activation (63), with continued studies ongoing.

Another significant hurdle in durable response to CAR therapy is limited survival of the CAR clone, and methods are under investigation to promote the persistence of the CAR T product, including optimizing manufacturing to increase selection of stem and central memory T cell subpopulations (64–66). As such, the bb21217 construct was created, which contains the same CAR design as ide-cel with addition of the PI3K inhibitor bb007, which increases the proportion of memory-type T cells (46). In preliminary data presented at the ASH 2021 national meeting from an ongoing phase 1 study (NCT03274219), 72 patients treated with bb21217 showed an OR rate of 69%, CR of 28%, and a median duration of response of 17 months. Toxicity was similar to ide-cel with a CRS incidence of 75% (4% grade ≥3) and neurotoxicity in 15% of patients (4% grade ≥3). Significantly, the CAR T clone was detectable in 81% and 60% of patients as 6 and 12 months, respectively (46). Additional methods to improve CAR T cell persistence and reduce functional exhaustion currently under development in preclinical models include the use of fully humanized variable chains to reduce immunogenicity and rejection of cells containing the CAR construct (67–70), combination costimulation with novel signaling pathways such as ICOS (71), cytokine manipulation to promote T cell activation and epitope spreading (72), combination therapy with immunomodulatory agents such as lenalidomide (73, 74), and blocking TGF-β responsiveness to inhibit tumor-induced immunosuppression (75).

Finally, underlying dysfunction of patient-derived T cells may limit clinical efficacy of the manufactured CAR T product. CAR T therapy is primarily used in patients with R/R disease, and autologous lymphocytes have therefore been exposed to the tumor’s immunosuppressive milieu as well as multiple lines of therapy prior to transducing the CAR construct (76). The composition of endogenous T cells used in manufacturing the CAR product, including the CD4:CD8 ratio and presence of central memory and stem cell memory T cells, has been shown to affect CAR T expansion, anti-tumor activity, and clinical response in myeloma and other B cell malignancies (77–79). One strategy to combat this is collection of T cells for CAR production at early stages of disease; in particular, one group showed T cells isolated from G-CSF-treated patients at the time of cell harvest for autologous stem cell transplant (SCT) showed appropriate expansion and anti-tumor activity *in vitro* and in mice (76).

In summary, the dramatic response rates observed following BMCA-directed CAR T cell therapy has added a vital tool to the treatment of patients with relapsed disease, and has had a meaningful impact on survival of patients with refractory myeloma. Limitations include lack of persistence of the CAR T cell population and the emergence of antigen negative variants. Strategies to overcome these mechanisms of resistance are a major focus of ongoing research and include the development of dual-targeting CARs and novel combinatorial approaches. In addition, the time needed to generate autologous CAR T cell products poses challenges to patients with aggressive advanced disease. Allogeneic CARs have the potential to overcome this limitation by providing an off the shelf product using allogeneic lymphocytes from healthy donors, as discussed below.

### Allogeneic CARs

Given the success of autologous CAR constructs, allogeneic CAR T cells are being explored, with multiple possible advantages over autologous cells. Allogeneic products address multiple logistical challenges with manufacturing and quality and can be immediately available as an “off-the-shelf” option for therapeutic use in patients with unstable disease (80). Additionally, allogeneic CARs rely on functional T cells from healthy donors, which may avoid the challenges discussed above of dysfunctional T cells derived from patients with R/R disease. In preclinical studies, BCMA-targeted CAR T cells from healthy donors showed increased T cell expansion and memory populations, increased cytotoxicity *in vitro*, and decreased checkpoint marker expression compared with cells generated from patients with R/R disease (81). The primary drawback of this approach is concern for rejection limiting the persistence of the allogeneic CAR product as well as possible GVHD, and prior attempts to reduce off-target effects have included deletion of the native T cell receptor (TCR) or use of non-αβ T cells (80). An ongoing phase 1 trial (NCT04093596) for R/R myeloma treated with ALLO-715, an allogeneic anti-BCMA CAR T cell with a disrupted TCR and CD52 gene to reduce risk of GVHD, was presented at the ASH 2021 national meeting. 26 patients treated at higher cell doses had an OR rate of 62% at median follow up of 7.4 months with CRS incidence of 52% (2% grade 3) in the overall cohort (82), though expanded studies with further follow up are likely needed to assess the incidence and severity of GVHD. While allogeneic CAR T cells show promise as an off-the-shelf immunotherapeutic for patients with MM, the risks of GVHD, complexities of gene editing including the risk of genetic mutations, and the host immune clearance of the allogeneic CAR product limiting expansion and persistence *in vivo* remain challenges to be overcome.

### Marrow-infiltrating lymphocytes

Marrow-infiltrating lymphocytes (MILs) are T cells directly exposed to malignant cells in the marrow microenvironment. These cells home to and survive in the marrow and contain a high proportion of tumor-specific cytotoxic lymphocytes (83). In an initial trial in MM, MILs were harvested and expanded ex vivo and reinfused in 25 patients after autologous SCT (84). Patients showed an OR rate of 54% with 27% CR, with a significant increase in PFS and OS in the subset of patients with significant disease reduction (84). Further studies are underway of MILs in combination with other standard and vaccine-based therapies.

### TCR-modified T cells

TCR-modified T cells (TCR T cells) are native T cells with edited TCRs to recognize a specific tumor antigen presented on the major histocompatibility complex (85). While CAR T cells exclusively bind cell surface proteins, TCR T cells allow for targeting of both intracellular and extracellular antigens. However, the major barrier to development is identification and generalizability of appropriate targets, as they are specific to each patient’s tumor antigens and HLA profile (85). In multiple myeloma, TCR T cells have been developed primarily targeting cancer testis antigens, such as NY-ESO-1, which is overexpressed in about one third of myeloma patients (86). A phase 1/2 trial of TCR T cells recognizing a shared peptide from NY-ESO-1 and the testis antigen LAGE-1 presented on HLA-A*0201 was administered to 20 patients after autologous SCT and showed an 80% response rate, 70% near CR at 100 days, estimated PFS of 19.1 months, and no evidence of CRS (87). Cytotoxicity in this model was shown to be further augmented *in vitro* with PD-1 blockade (88), and a phase 1 study of an NY-ESO-1/LAGE-1 T cell with CRISPR deletion of the native TCR and endogenous PD-1 gene showed initial feasibility (89). However, despite the lack of CRS, off-target toxicity of TCR T cells towards similar epitopes from other proteins as well as on-target/off-tumor toxicity directed against shared tumor antigens in other tissues remains a significant concern. Notably, an engineered affinity-enhanced HLA-A*01-restricted MAGE-A3-targeted T cell induced cardiogenic shock and death from myocardial infiltration in both treated patients in an initial study, likely due to cross-reactivity with epitopes from the native cardiac protein titin (90). While TCR T cells may represent a promising avenue to target an expanded set of intracellular antigens in a subset of patients with specific HLA phenotypes, further work is needed to appropriately identify effective targets while limiting alloreactivity towards normal tissues.

### NK cells and NK CARs

Adoptive NK cell therapy represents an additional promising avenue for anti-tumor immunity. NK cells possess multiple advantages over T cells, including cytotoxic activity without prior antigen exposure or HLA restriction and reduced risk of GVHD. In a phase 1 study, 12 myeloma patients received cord blood-derived NK cells in association with autologous SCT. The study showed an OR of 83% and near CR in 67% of the cohort, detectable NK cells *in vivo* at 6 months, and no evidence of GVHD (91), with a phase 2 study underway (NCT01729091). CRISPR-edited NK cells with reduced expression of the inhibitory KLRC1 locus showed improved anti-myeloma cytolytic activity *in vitro*, and further study is needed for the clinical utility of ex vivo NK cell engineering in myeloma (92). Additionally, CAR NK cells are being developed with modified targeted antibodies bound to NK cell signaling proteins. Further, NK cells from an allogeneic donor may allow for rapid production of an off-the-shelf CAR NK cell product, do not need to be individualized for each patient, and do not require CRISPR gene editing as do allogeneic CAR T cells in order to mitigate the risk of GVHD (93, 94). In murine models, CAR-NK cells targeting CD138 and CS-1 have showed some efficacy, with further preclinical work needed (95, 96).

## Checkpoint inhibitors

Immune checkpoint blockade through the PD-1/PD-L1 or CTLA4 axes has had remarkable clinical efficacy in other malignancies in reducing immune dysregulation in the tumor microenvironment and re-activating native adaptive immunity. PD-L1 is highly expressed on malignant plasma cells and is associated with resistance to therapy and relapse, suggesting it may be an important pathway in mediating immune dysregulation (7, 97–99). However, pharmacologic PD-1 blockade in R/R myeloma did not show clinical benefit when used as monotherapy (100). Additionally, two phase 3 randomized trials of PD-1 blockade combined with immunomodulatory agents (lenalidomide/pomalidomide) were stopped prematurely due increased toxicity without a signal of improved efficacy, and there are ongoing concerns about the safety of checkpoint inhibitors in MM (101, 102). Further work is needed to define the potential therapeutic role of checkpoint inhibitors in myeloma, including in combination with other immune-based therapies such as vaccines (103, 104) and adoptive cell therapy (60, 105, 106).

## T cell engagers

To reduce immune evasion and dysregulation, bispecific T cell engagers (BiTEs) have been developed in multiple hematologic malignancies. These antibodies bind a specific tumor antigen as well as CD3 to induce colocalization of T cells with neoplastic cells in the context of T cell activation. BiTE antibodies utilize the native T cell repertoire and can induce a polyclonal response with expansion of memory populations; however, they are therefore dependent on native T cell quality and function, which may be impaired in the setting of malignancy (9).

In MM, BiTE development has centered on BCMA as a plasma cell marker. There are multiple CD3/BCMA constructs currently under investigation, with some early evidence of efficacy and durable response in phase 1 trials (107–112), and multiple phase 1/2 trials ongoing (NCT03145181, NCT03287908, NCT03933735, NCT03486067, NCT03761108). In a study of the AMG 420 compound in 42 patients with R/R disease, the OR rate was 70% with median response of 9 months in patients who received the target dose, though with 48% of patients developing grade 3 adverse events (107). A phase 1/2 trial of teclistamab in 165 patients showed an OR of 63%, CR of 39%, and median PFS of 11.3 months at median follow up of 14.1 months. Patients had a 72% incidence of CRS (0.6% grade ≥3) and 15% neurotoxicity (0 grade 3) (113). As noted above, γ-secretase inhibitors increase membrane BCMA expression, and they have been shown to augment the efficacy of an anti-BCMA BiTE *in vitro*, suggesting they may be effective as complementary agents (114). Multiple other BiTE targets are under investigation in preclinical and early clinical studies, including GPRC5D (NCT03399799) (115), FcRH5 (NCT03275103) (116), and CD38 (117). BiTEs represent a highly exciting therapy, as evidenced by promising response rates in the setting of advanced myeloma. Understanding how to best incorporate BiTE therapy earlier in the disease course and how to sequence various BCMA directed therapies is an area of ongoing investigation. Importantly, using anti-BCMA BiTEs as a partner in combination therapy, including with CAR T cells, immunomodulatory drugs, and vaccines, has the potential to further improve outcomes for patients with MM.

## Vaccine therapy

Cancer vaccine therapy utilizes peptide or cell-based methodologies to facilitate presentation of tumor-specific antigenic targets with the goal of activating and expanding cytotoxic T cells to target malignant cells. Vaccines have the potential to induce a polyclonal response to capture the heterogeneity of the tumor as well as induce a memory response to mitigate risk of disease relapse (118, 119). Challenges in vaccine development and clinical efficacy include identification of appropriate antigenic targets that are sufficiently immunogenic and specific to the malignant clone as well as effective presentation by antigen presenting cells in the context of costimulation (119).

Early efforts in immunization in myeloma have focused on peptide-based vaccine strategies targeting upregulated or aberrantly expressed proteins by the myeloma clone to induce tumor-specific immunity, with selected trials highlighted in **Table 2**. These vaccines have generally induced T cell and interferon responses but have not markedly improved clinical outcomes. An anti-MUC1 signal peptide vaccine, ImMucin, led to myeloma-specific immunity and at least disease stabilization in most patients (120). RHAMM-R3 peptide vaccination resulted in immunologic and biochemical responses in patients with myeloma overexpressing RHAMM (121, 122). Vaccination against the anti-apoptotic Bcl-2 family proteins generated immune responses in relapsed myeloma patients in a phase 1 clinical trial (123). Idiotypic DNA vaccines targeting the myeloma-specific immunoglobulin were also noted to engender immune responses in a phase 1 clinical trial (124). A recent phase 2 randomized study of an idiotype vaccine paired with adoptive transfer of ex-vivo expanded, vaccine-specific autologous T cells showed increased markers of functional immune activation and memory generation, though without significant difference in CR or 3-year PFS (125). Additionally, to decrease the risk of off-target toxicity, cancer-testis antigens such as NY-ESO-1 and MAGE-A3, proteins normally restricted to fetal development or germ cells but aberrantly re-expressed in malignant cells, have been targeted, with immunization leading to durable immune responses in proof-of-concept studies (129, 130).

**Table 2:** Selected clinical trials of peptide or pulsed/loaded dendritic cell vaccines in myeloma.

Phase	Pts	Target	Immune Response	Clinical Response	Ref.
1/2	15	MUC1	T cell and antibody response and increased IFNγ in all patients	73% stable/improved disease, median PFS 17.5 mo.	(120)
1	7	RHAMM-R3	CD8 T cell response in 71% of patients	Reduced free light chains in 43% of patients	(121, 122)
1	7	Bcl-2 family	T cell response in all 6 patients with at least 2 vaccinations	No change	(123)
1	14	Idiotype protein linked to FrC of tetanus toxin	T cell response to idiotype in 29% of patients	Ongoing CR/PR in 79%, median time to progression 38 mo.	(124)
2	16	Idiotype protein linked to KLH	Upregulated immune reconstitution genes in T cells of treated patients	CR 50% vs 30% (p=.22), 3-yr PFS 56% vs 59% (p=.32) in treated (n = 16) vs control (n = 20) patients	(125)
1/2a	22	XBP1, CD138, CS1 (PVX-410)	CD8 T cell and IFNγ response in 95% of patients, further increased in combination with lenalidomide	20% partial or minimal response, 50% stable disease	(126)
2	27	Idiotype-pulsed DCs	Not evaluated	OS 5.3 vs 3.4 yrs (p=.02), no change in median PFS in treated patients vs unmatched cohort from similar period	(127)
1	9	DCs loaded with irradiated myeloma cells	T cell or IFNγ response in 78% of patients treated at higher dose	11% minimal response, 56% stable disease	(128)

FrC, fragment C; KLH, keyhole limpet hemocyanin; DC, dendritic cell; PFS, progression-free survival; CR, complete response; PR, partial response; OS, overall survival.

Despite some immune efficacy noted above, there are multiple major limitations of antigen peptide-based vaccination, including potential resistance by antigenic escape and downregulation of target antigens. To combat this, multiepitope vaccines have been trialed, and one such strategy targeting XBP1, CD138, and CS1 peptides in smoldering myeloma induced a robust myeloma-specific immune response *in vitro* (131). In a nonrandomized clinical trial, patients demonstrated a vaccine-specific T cell response that was further enhanced when administered in conjunction with lenalidomide, though with few significant clinical responses (126). Another concern with peptide vaccines is limited immunogenicity of self-antigens despite overexpression by the tumor given thymic deletion of T cells with high affinity for these antigens during development (132). As a note, this decreased activity of the native T cell repertoire in response to self-antigens is likely why vaccination against cancer testis antigens NY-ESO-1 or MAGE-A3 is well tolerated, while infusion of ex-vivo engineered TCR T cells targeting these same antigens led to significant alloreactivity and toxicity, as discussed above. Alternatively, novel protein sequences and foreign antigens created by tumor-specific mutations, or neoantigens, may induce robust T cell recruitment and anti-tumor immune response. Interestingly, neoantigen burden in myeloma has been found to correlate with inferior survival and associated upregulation of T-cell suppression pathway genes, creating an opportunity for neoantigen-based vaccination as a vehicle for activation of suppressed T cells and host anti-tumor immunity (133–135). While neoantigens may be patient or tumor-specific, shared neoantigens can be identified from common oncogenic driver mutations that can be feasibly targeted by vaccines. In myeloma, common shared neoantigens such as PKD1, PRKDC, and NRAS are being further characterized in the laboratory, and in preclinical studies, a neoantigen-based vaccine targeting MOPC315 has been shown to induce anti-tumor immunity (136, 137). Further research is needed in neoantigen identification and immunogenicity to determine the potential of neoantigen-based vaccines as a therapeutic strategy in myeloma.

In addition to selecting an optimal tumor-specific antigen, promoting a method of antigen uptake and presentation is key in inducing robust and durable anti-tumor immunity and mitigating the effects of immune tolerance within the tumor microenvironment. As such, a variety of DC-based vaccines strategies have been developed to ensure antigen presentation in the context of DC-mediated costimulation. In clinical trials, these strategies most commonly involve reinfusion of ex vivo DCs pulsed with tumor antigens, tumor cell lysate, or apoptotic bodies, with the goal of DC loading with varied tumor antigens to reduce risk of antigenic escape (138, 139). Idiotype-pulsed DCs were associated with prolonged survival following autologous SCT (127). Additionally, a study using DCs loaded with irradiated autologous MM cells was well tolerated and resulted in immune responses and disease stabilization in a phase 1 trial of patients with R/R myeloma (128).

An additional approach is manipulation of patient-derived tumor cells to express GM-CSF, thereby promoting DC migration to the site of vaccination. This strategy, also known as GVAX, was initially applied in acute myeloid leukemia (140, 141). The GVAX platform has been employed in combination with lenalidomide in myeloma patients in near complete remission. In a proof-of-principle study, over 50% of treated patients reached CR, with median PFS not yet reached at median follow up of 5 years and with persistent immunologic responses in all patients (142).

Our group has developed a personalized vaccine strategy wherein autologous DCs are fused with patient-derived tumor cells, which creates a hybridoma expressing a wide variety of tumor-specific neoantigens in the context of increased costimulation. In a phase 1 trial evaluating this DC/myeloma fusion vaccine, vaccination was well tolerated and resulted in expansion of CD4 and CD8 myeloma-reactive T cells and disease stabilization in the majority of patients (143). In a subsequent phase 2 trial following autologous SCT, vaccination significantly increased CD4 and CD8 myeloma-specific T cells, with a CR rate of 47% and a 2-year PFS of 57% (144). In light of these encouraging results, a randomized multicenter trial of the DC/myeloma fusion vaccine versus lenalidomide maintenance alone after autologous SCT is currently in progress (NCT02728102).

## Conclusions

Immune-based therapies have demonstrated exciting results in patients with MM. Ongoing areas of research focus remain understanding and overcoming mechanisms of resistance, optimizing combinatorial approaches, and identifying biomarkers of resistance and response. The potency of CAR T cell therapy has been demonstrated in the setting of advanced disease, and it is currently being investigated in the setting of high-risk disease in the newly diagnosed setting. In newly diagnosed disease, the current treatment landscape for myeloma is such that with immunomodulatory agents, proteasome inhibitors, and CD38-targeting antibodies, one can achieve deep responses. Incorporating immune based therapy has the potential to eradicate minimal residual disease to promote immune surveillance and protect from relapse. Understanding and addressing mechanisms of resistance and biomarkers of response will be critical toward designing the appropriate combinatorial approach, choosing the optimal sequence of therapy, and ultimately, developing a curative therapeutic approach to the treatment of MM.

## Author contributions

All authors listed have made a substantial, direct, and intellectual contribution to the work and approved it for publication.

## Conflict of interest

Author JR has the following conflicts of interest: Attivare Therapeutics- consultant; Bioclinica- consultant; Parexelconsultant Imaging endpoints- consultant: BMS- advisory board, research funding; Karyopharm-DSMB; Celgene - research funding; Sanofi - research funding.

The remaining authors declare that the research was conducted in the absence of any commercial or financial relationships that could be construed as a potential conflict of interest.

## Publisher’s note

All claims expressed in this article are solely those of the authors and do not necessarily represent those of their affiliated organizations, or those of the publisher, the editors and the reviewers. Any product that may be evaluated in this article, or claim that may be made by its manufacturer, is not guaranteed or endorsed by the publisher.
